# Robotic-assisted total knee arthroplasty improves the rotational mismatch between femoral and tibial components, but not the forgotten joint score 12: a single-center retrospective cohort study

**DOI:** 10.1186/s40634-023-00705-w

**Published:** 2023-12-08

**Authors:** Ayakane Yamamoto, Takao Kaneko, Kazutaka Takada, Shu Yoshizawa

**Affiliations:** Ichinomiya Onsen Hot Hospital, Adult Reconstruction Center, 1745 Tsuboi, Ichinomiyacho, Fuefuki-City, Yamanashi 405-0077 Japan

**Keywords:** Image-free robotic assisted total knee arthroplasty, Conventional total knee arthroplasty, Three-dimensional computed tomography, Patient reported outcome measurements, Forgotten joint score-12, Rotational mismatch

## Abstract

**Purpose:**

The primary aim of this study was to compare postoperative short-term patient reported outcome measurements (PROMs) and rotational mismatch between femoral and tibial following conventional jig-based total knee arthroplasty (Conv-TKA) versus robotic-assisted TKA (RA-TKA) using three-dimensional computed tomography (3DCT) measurements.

**Methods:**

This retrospective, consecutive case–control trial included 83 patients with varus osteoarthritis of the knee undergoing Conv-TKA versus RA-TKA using bi-cruciate stabilized TKA. The rotational mismatch of the femoral and tibial components between the two groups were compared using 3DCT measurements. PROMs (2011 Knee Society Score (KSS), forgotten joint score-12 (FJS-12), patella score were compared in patients between 1 and 2 years postoperatively.

**Results:**

The two groups did not exhibit significant differences in any of the following preoperative factors: age at surgery, body mass index (BMI), preoperative range of motion (ROM), hip-knee-ankle (HKA) angle. There were no significant differences in postoperative HKA angle and tibial rotation angle, the absolute values of the femoral rotational angle and rotational mismatch were significantly smaller in the RA-TKA group than in the Conv-TKA group (both *p* < 0.01). Neither Postoperative PROMs (2011 KSS: pain, patient satisfaction, patient expectation, advanced activities score) nor patella score differed significantly between the groups, but FJS-12 was significantly better in the Conv-TKA group than in the RA-TKA group (*p* < 0.01).

**Conclusions:**

RA-TKA did not improve FJS-12 compared to Conv-TKA, but did result in more accurate rotational alignment of femoral component and rotational mismatch between the femoral and tibial components.

**Level of evidence:**

IV.

## Introduction

Total knee arthroplasty (TKA) is one of the most successful treatments for reducing pain and improving function in patients with end-stage knee osteoarthritis. Some national registries with long-term follow-up data have shown that TKA implants have a survival rate of over 90% at 10 years [1–4]. However, 15 to 20% patients are not satisfied with their new joint, and up to one-third of patients report that their joint does not feel normal after TKA [5, 6].

The keys to success in TKA are accurate osteotomy, mechanically alignment (MA) of the components, and good soft tissue balance. In 1976, Insall J et al. [7] first coined the terms flexion gap and extension gap. To achieve balance between these two gaps, they advocated the classic method of bone resection and aforementioned soft tissue releases.

In recent years, with advances in preoperative three-dimensional computed tomography (3DCT) planning [8], patient-matched-instruments [9], computed assisted surgery, and manual and digital soft tissue balancing tools, the first three elements can now be achieved more accurately. Manual soft tissue balancing tools such as balancer are difficult to use for freehand osteotomies where joint pressures are fine-tuned for measurement after osteotomy, while a digital soft tissue balancing tool　using the VERASENSE® sensor-guided balancing technology (Orthosensor Inc) did not improve range of motion (ROM) and patient reported outcome measurements (PROMs) at 2 years postoperatively, despite increased operative time and cost, and is not recommended for routine use [10].

Recently, a variety of semiactive robotic-assisted (RA) systems for orthopaedic surgery (with or without images, and using different cutting systems and planning methods) have been promoted worldwide. As for component accuracy, RA surgical techniques have given surgeons intra-operative options to improve accuracy [11–14]. One of the most important features is that some RA systems show data about the gaps all over ROM.

Previous reports have improved the accuracy of implant placement but have not evaluated the rotational mismatch between femoral and tibial components in RA surgical technique.

Regarding patient reported outcome measurements (PROMs), a meta-analysis of outcome data demonstrated that robot-arm TKA (MAKO: robotic interactive orthopedic arm system, Stryker, Fort Lauderdale, Florida, USA) resulted in significantly better PROMs than conventional jig-based (Conv)-TKA after short- to mid-term follow up [15]. In contrast, RA-arm TKA resulted in a qualitatively higher Knee Society (KS) composite function score at 1 year postoperatively than Conv-TKA, although the difference was not statistically significant [16]. On the other hand, two studies of an image-free handheld RA-TKA (Blue Belt Navio surgical system., Navio, Smith & Nephew, Plymouth, MN, USA) found no significant differences between RA-TKA and Conv-TKA at 12 or 20 months after surgery for any of the following PROMs; the Short Form-12 score, Westren Ontario and MacMaster Universities Osteoarthritis Index (WOMAC) score and Knee Society Score (KSS) functional score [17, 18]. However, these studies did not use uniform prosthetic designs, and PROMs such as patient satisfaction and patient expectations were not adequately evaluated.

The purpose of this study was to compare the RM between femoral and tibial component and PROMs of patients who underwent an image-free handheld RA-TKA and Conv-TKA using bi-cruciate stabilized TKA (BCS) (Journey II BCS; Smith & Nephew. Inc. Memphis, TN, USA).

We hypothesized that RA-TKA improves RM and short-term postoperative PROMs more than Conv-TKA.

## Methods

The study design was approved by the Ethics Review Committee (21010). All patients who participated provided written informed consent. Between 2019 and 2020, this retrospective case control study enrolled consecutive 53 patients who underwent TKA using an image-free handheld RA surgical system (Robot group) and between 2018 and 2019, this retrospective case control study enrolled consecutive 41 patients who performed TKA using a conventional manual surgical procedure (Manual group). The patients were not randomized and the same surgeon used the BCS prosthesis in both groups. The inclusion criteria were substantial pain, loss of function due to varus-type osteoarthritis of the knee and availability of complete data over 1 year of postoperative follow-up. Exclusion criteria included valgus-type osteoarthritis of the knee, rheumatoid arthritis, previous hip or knee arthroplasty surgery, severe bony defects requiring bone graft or augmentation, revision TKA, lumbar region problems, and active knee joint infection.

Preoperative patient demographics including age, gender, body mass index (BMI), and operation time, were closely matched in both groups. Matching criteria are summarized in Table 1.

**Table 1:** Preoperative patient demographics data

	**Robot group (*N* = 53)**	**Manual group (*N* = 41)**	***p* value**
**Age (year)**	76.7 (SD 6.7; 51 ~ 90)	75.3 (SD 8.8; 58 ~ 92)	n.s
**Gender (male: female)**	15: 38	7: 34	n.s
**BMI (kg/m**^**2**^)	25.4 (SD 4.0; 20.4 ~ 51.5)	24.6 (SD 2.1; 19.3 ~ 27.8)	n.s
**F/U period (months)**	15 (SD 5.2; 12 ~ 24)	14 (SD 4.6; 12 ~ 24)	n.s

Mean, standard deviation, and range were provided

*BMI* Body mass index, *F/U* Follow up, *SD* Standard deviation,* n.s* Not significant

### Surgical procedure

**Fig. 1:**
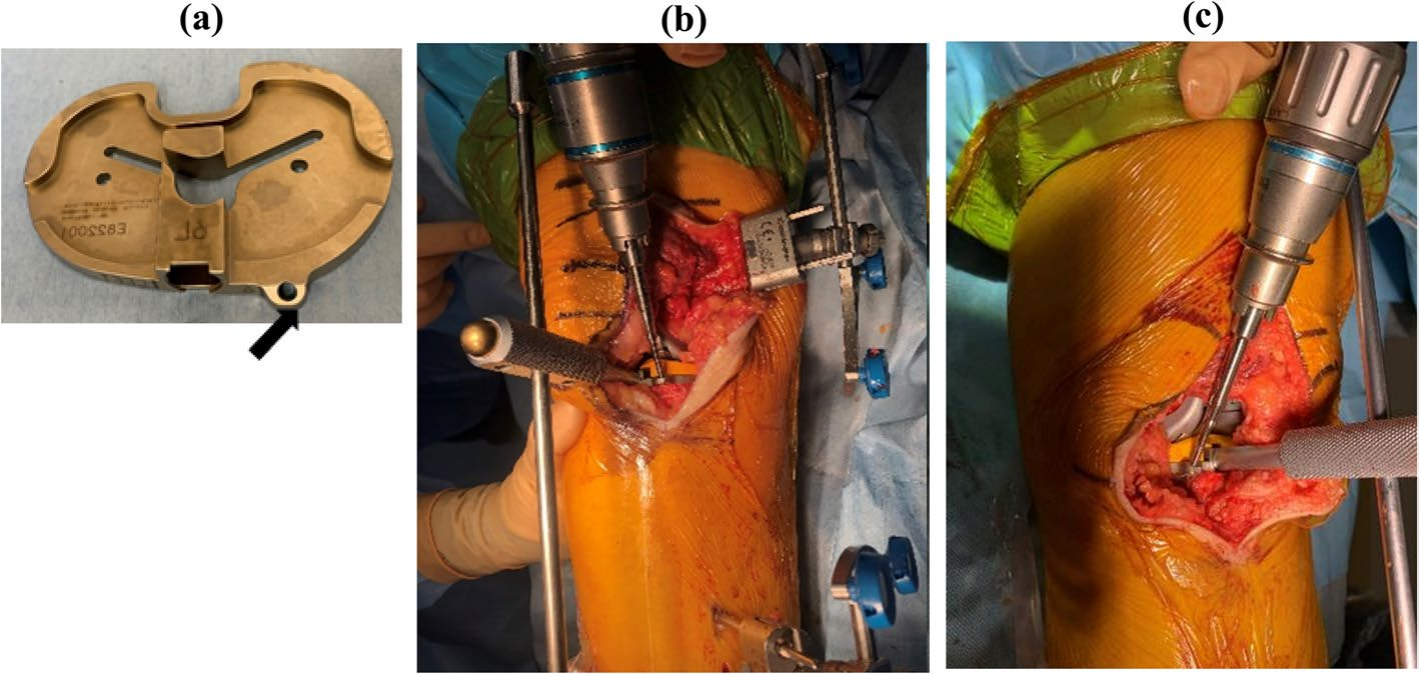
We created a unique pinhole (black arrowhead) in the anteromedial base plate to perform this accuracy (**a**). The tibial fixation to determine the rotational alignment was similar in both Robot (**b**: right knee) and Manual groups (**c**: left knee). The determination of the rotational alignment of the tibial component used the range of motion (ROM) method, depending on the rotational alignment of the femoral component and soft tissue balancing [18, 23, 24]

Conv-TKAs were performed using conventional manual surgical procedures with the measured resection technique. After inflating a tourniquet to 300 mmHg at the beginning of the procedure, a subvastus arthrotomy was performed. A distal femoral osteotomy was performed at the valgus angle of the femur; this angle was measured between the mechanical axis and the anatomical axis using an intramedullary resection guide during preoperative 3DCT planning of the entire lower extremity. Rotational alignment of femoral component was aligned parallel to the surgical epicondyle axis and perpendicular to the whiteside line. An anterior or posterior referencing technique was used for the anterior and posterior femoral cut. The size of the femoral component was determined based on the anteroposterior length of the femur. The medial joint gap in extension and at 90 degrees of flexion was kept constant, regardless of the size selected or the external rotation angle. If the flexion gap was loose, the femoral sizing guide could be moved posteriorly so that the anterior notch did not occur in the frontal cortical bone. If the flexion gap was too tight, the guide can be moved anteriorly. The differences between the extension gap and the flexion gaps of the medial compartment were reduced as much as possible. An extramedullary resection guide was used for proximal tibial osteotomy. The angle of the osteotomy was perpendicular to the mechanical axis, and the tibial posterior tilt had an 85-degrees orientation. The landmark used to determine the rotational alignment of the tibia was Akagi’s line, defined as a straight line from the middle of the posterior cruciate ligament and the medial border of the patellar tendon attachment site [19]. Furthermore, Akagi's Line was perpendicular to the surgical epicondylar axis with the knee in extension [20], which is important because the BCS-TKA results in a guided motion designed to induce medial pivot movement. Finally, the position of the tibial component was fine-tuned and determined by the ROM method [12, 21, 22] (Fig. 1a, c). Therefore, excessive external rotation alignment of the femoral component was avoided due to rotational mismatch.

The patella was resurfaced. In medial ligament balance in extension, we released the deep medial collateral ligament (d-MCL) and removed osteophytes within 1 cm of the joint line against osteophytes attached to the d-MCL, which affect the extension gap. The superficial layer of the MCL, semimembranosus, and posterior oblique ligament were not released.

Image-free handheld RA surgeries were performed using the Navio RA system [12, 18, 21, 25–27]. After inflating a tourniquet to 300 mmHg at the beginning of the procedure, a subvastus arthrotomy was performed. Planning of prosthesis position and bone resection was determined intraoperatively considering soft tissue balance. Osteophytes on the femur and tibia were resected. The tracker fixation of the femur was proximal and anterior to the medial epicondyle and mid-shaft femur, while that of the tibia extended from the anteromedial tibia to 6 cm from the wound using bury pin threads (4.0 mm). Landmark registration, ROM, varus-valgus laxity mapping, and anatomy of the femoral condyle and tibial plateau was mapped by ‘‘painting” the surfaces with an optical probe. A virtual model of the knee was thus created. The surgeon intraoperatively determined the volume of bone removal and planned the prosthesis size, alignment, and position.

Based on previous reports using a balancer, the femoral rotation position was determined so that the medial joint gap was almost constant from extension to 90 degrees of flexion and the lateral joint gap was loose at 90 degrees of flexion [21, 28]. Arthritic cartilage and bone were then methodically removed using the handheld sculptor. The fixation of the tibial component to determine the rotational alignment was similar in the Manual group, regardless of RA surgery (Fig. 1a, b). In this technique, the rotational alignment of the tibial component is determined through conformity to the femoral component when the knee is put through a series of full flexion–extension cycles [24]. The RA surgical technique continuously tracked the position of the patient’s lower limb and the progress of bone resection using a navigation system camera [12, 18, 21, 25–27].

The drainage tube was removed and physical therapy was initiated in both the Robot and Manual groups at first day after surgery. Full weight-bearing was not restricted and patients were allowed to walk with or without assistive devices. All the patients followed the same postoperative rehabilitation protocol.

### Pre- and postoperative three- dimensional computed tomography images

The preoperative plans were developed using 3DCT data of the entire extremity in all cases. Using reference points, preoperative CT images were automatically fused to postoperative images by matching bone surfaces. After matching, the femoral-tibial component template from a computer- aided design model was manually superimposed on the implant image to match their contours.

**Fig. 2:**
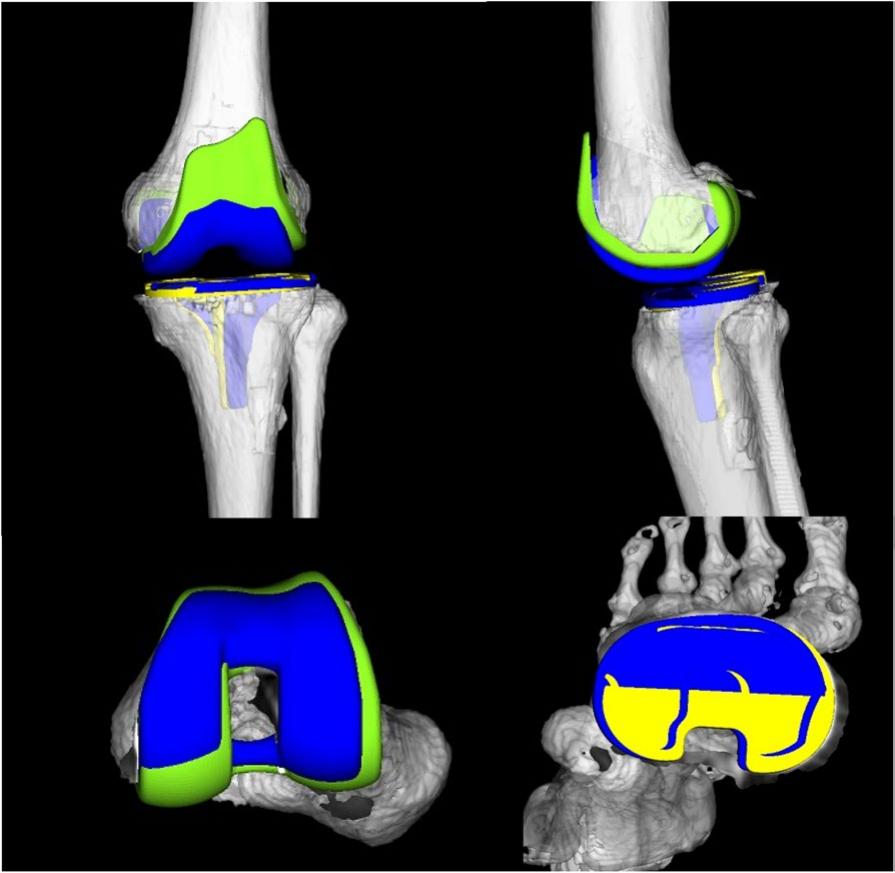
The preoperative three-dimensional computed tomography image (3DCT) plan and a postoperative 3DCT image are shown. 3D computer-aided design data of femoral and tibial components were fit to the 3DCT image using six parameters, specifically the coronal, sagittal, and axial alignment of the femoral and tibial prostheses. The blue components indicate the preoperative plan image; the green and yellow components indicate the postoperative component

Based on the positioning of the templates, the coronal and axial alignments of both components were measured with reference to the coordinate system using ZedView (ZedKnee; LEXI Co., Ltd., Tokyo, Japan). Postoperative CT scans were obtained at 4-weeks in both groups. The postoperative hip-knee-ankle (HKA) angle was also measured as the leg axis between the anatomical axis of the femur and tibia in the coronal plane defined by the coordinate systems. The postoperative 3DCT images of the femur and tibia were superimposed onto those of the preoperative 3DCT plan using ZedView software (Fig. 2) [12, 22, 26, 27].

**Fig. 3:**
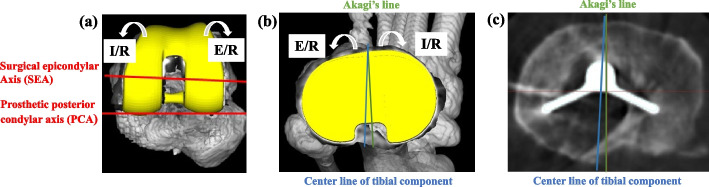
The postoperative three-dimensional computed tomography image (3DCT) image are shown. **a** Femoral component rotational angle. Axial 3DCT image of the left femur. The surgical epicondylar axis (SEA) connects the lowest point of the medial epicondyle to the midpoint of the lateral epicondyle. The prosthetic posterior condylar axis (PCA) connects the medial and lateral prosthetic posterior condylar surfaces. The femoral component rotational angle was defined as the angle between the SEA and the PCA. **b**, **C** Tibial component rotational angle. Axial 3DCT image of the left tibia. Akagi’s line connects the center of the posterior cruciate ligament and the medial border of the tibial tuberosity. The tibial component rotational angle was defined as the angle between the centerline of the tibial component and Akagi’s line. A positive value represents external rotation, and a negative value represents internal rotation of the femoral and tibial components. The absolute value of the angular divergence of the femoral component relative to the tibial component is defined as the rotational mismatch [27]. E/R; external rotation, I/R; internal rotation

Rotational alignment of the femoral and tibial components relative to the bone landmarks and mismatch between the components were measured using 3DCT images. The femoral component rotational angle (+ : external rotation, -: internal rotation). Figure 3 was defined as the line perpendicular to the surgical epicondylar axis, the line between the lowest point of the medial epicondyle and the midpoint of the lateral epicondyle [29]. The tibial component rotational angle (+ : external rotation, -: internal rotation). Figure 3 was defined as Akagi’s line, the line between the center of the posterior cruciate ligament and the medial border of the tibial tuberosity [19]. On each postoperative 3DCT image, a positive value represents external rotation of the femoral and tibial components, while a negative value represents internal rotation. The absolute value of the angular divergence of the femoral component relative to the tibial component is defined as the rotational mismatch (Fig. 3) [30].

A previous study showed that interclass correlation coefficients for 3DCT evaluations in the coronal, sagittal, and axial planes were 0.901, 0.899, and 0.881 for the femur and 0.924, 0.911, and 0.899 for the tibia, respectively. Intraclass correlation coefficients for the coronal, sagittal, and axial planes were 0.956, 0.903, and 0.878 for the femur and 0.918, 0.815, and 0.896 for the tibia, respectively [12, 27].

### Patient reported outcome measurements

PROMs were assessed 1 and 2 years postoperatively using four sections (symptom, patient satisfaction, patient expectation and advanced activities) of the 2011 KSS [31], 12 items of the Forgotten Joint Score (FJS-12) [32], and the patella score [33].

### Statistical analysis

Means and standard deviations were used to describe the data. Student’s t-test or the Wilcoxon nonparametric test were implemented. Categorical variables were compared using the Fisher exact test. All statistical analyses were performed with SPSS version 24.0 software (SPSS Inc, Chicago, IL). Statistical significance was set at a *p* value of less than 0.01. A power analysis using the G*Power 3 analysis program [34] was performed using an α error of 0.01 and a 1 − β error of 0.80 (Type II error is no more than 20%) to compare the means between the two groups, and it indicated that a sample of 26 knees was sufficient to detect differences between the RA and Manual groups.

## Results

Preoperative patient demographics datas are shown in Tables 1 and 2. There were no significant differences between the two groups in terms of age, gender, BMI or follow up duration (Table 1). There were no significant differences between the two groups in terms of preoperative ROM, 1989 Knee Society Knee and Function score, and HKA angle (Table 2). The Robot group presented a significantly smaller rate of outliers for the femoral axial alignment than the Manual group (*p* < 0.01, Table 3). The absolute values in rotational mismatch were less in the Robot group than Manual group (*p* < 0.01, Table 3).

**Table 2:** Preoperative range of motion (ROM), Knee society 1989 (knee and function score), and hip-knee-ankle (HKA) angle

	**Robot group (*N* = 53)**	**Manual group (*N* = 41)**	***p* value**
**Preoperative**			
**Extension angle (°)**	-6.3 (SD 3.9; -12 ~ -5)	-5.4 (SD 5.6; -20 ~ -5)	n.s
**Flexion angle (°)**	113.9 (SD 6.3; 95 ~ 120)	116.9 (SD 7.4; 100 ~ 130)	n.s
**1989 Knee society**			
**Knee score**	16.8 (SD 13.2; 2 ~ 40)	15.8 (SD 10.7; 0 ~ 40)	n.s
**Function score**	56.5 (SD 15.6; 5 ~ 90)	47.5 (SD 25.2; 5 ~ 90)	n.s
** HKA angle (°)**	187.3 (SD 4.1; 180 ~ 200)	187.3(SD 4.6; 180 ~ 201)	n.s

*Mean* Standard deviation, *SD* Standard deviation, and range were provided

**Table 3:** The values in the femoral and tibial component rotational angle and absolute values in the rotational mismatch of the femoral and tibial component in postoperative 3DCT image

	**Robot group (*N* = 53)**	**Manual group (*N* = 41)**	***p* value**
**Femoral component rotational angle (°)**	2.4 (SD 2.6; -3.5 ~ 9.0)	9.2 (SD 2.6; 3.8 ~ 13.7)	< 0.01
**Tibial component rotational angle (°)**	0.6 (SD 6.5; -9.6 ~ 17)	1.5 (SD 7.6; -9.9 ~ 19)	n.s
**Rotational mismatch (°)**	4.9 (SD 4.4; 0.1 ~ 15.2)	9.3 (SD 6.2; 0.4 ~ 25.6)	< 0.01

A positive value represents external rotation, and a negative value represents internal rotation of the femoral and tibial components. The value of the angular divergence of the femoral component relative to the tibial component is defined as the rotational mismatch (Fig. 2) [27]

*3DCT* three-dimensional computed tomography measurements, *Mean* Standard deviation, and range were provided, *SD* Standard deviation, *ns* not significant

Postoperative ROM, 2011 KSS subscale scores (symptom, patient satisfaction, patient expectation, and advanced activities), FJS-12 score and patella score are shown in Table 4. Postoperative PROMs (symptom, patient satisfaction, patient expectation, advanced activities) and patella score were not significantly different between the two groups, but FJS-12 score was significantly higher in the Manual group than in RA group (*p*< 0.01).

**Table 4:** Postoperative range of motion (ROM), postoperative 2011 Knee society score (symptom, patient satisfaction, patient expectation and activity) and Patella score

	**Robot group (*N* = 53)**	**Manual group (*N* = 41)**	***p* value**
**Postoperative**			
**Extension angle (°)**	0.1 (SD 0.7; 0 ~ 5)	0.49 (SD 1.5; 0 ~ 5)	n.s
**Flexion angle (°)**	124 (SD 10.6; 90 ~ 146)	123.6 (SD 7.8; 105 ~ 138)	n.s
**2011 Knee society score**			
**Symptom**	18.8 (SD 4.0; 6 ~ 25)	19.9 (SD 4.7; 8 ~ 25)	n.s
**Patient satisfaction**	26.2 (SD 5.7; 16 ~ 40)	27.7 (SD 8.1; 16 ~ 40)	n.s
**Patient expectation**	9.7 (SD 2.1; 5 ~ 14)	10.4 (SD 2.9; 5 ~ 15)	n.s
**Activity**	57.8 (SD 14.5; 22 ~ 87)	64.1 (SD 18.6; 16 ~ 89)	n.s
**FJS-12**	52.2 (SD 18.8; 15 ~ 100)	63.5 (SD 20.1; 15 ~ 100)	< 0.01
**Patella score**	23.9 (SD 4.0; 13 ~ 30)	23.2 (SD 3.2; 18 ~ 28)	n.s

*Mean* standard deviation, *SD* Standard deviation, and range were provided, *activity* advanced activities, *FJS-12* Forgotten joint score 12

## Discussion

The main finding of the present study was (1) RA-TKA reduced the outliers in terms of rotational alignment of the femoral prosthesis and rotational mismatch between femoral and tibial components, compared to Conv-TKA, but (2) RA-TKA did not show better short-term postoperative improvement than Conv-TKA with respect to the FJS-12.

This is the first study to compare an image-free handheld RA-TKA and Conv-TKA in term of rotational mismatch using pre- and postoperative 3DCT data and detailed PROMs such as the 2011 KSS, FJS-12, and patella score. Previous reports comparing imageless RA TKA and Conv-TKA [17, 18] included Journey II BCS and Legion Posterior-Stabilized prostheses with different prosthetic designs in both groups. Regarding PROMs, only WOMAC, SF-12, 1989 Knee society functional score and 2011 Knee society score have not been examined. It is a strong point that this study was able to evaluate the postoperative alignments accurately with the 3DCT measurements using CAD software.

As for RA-TKA component accuracy attained using Navio, Bollars et al. reported that RA-TKA allowed the surgeon to accurately achieve the planned mechanical axis with significantly fewer outliers than Conv-TKA [35], and Navio TKA resulted in accurate alignment in more than 93% of cases [36]. Both Navio and CORI TKA (CORI surgical system., Smith & Nephew, Plymouth, MN, USA) demonstrated high levels of component alignment accuracy and ease of use [37]. All of these reports assessed the component accuracy of RA procedures using 2D radiographic measurements, and did not evaluate rotational alignment or rotational mismatch between femoral and tibial components. Several studies reported that despite the success of computer navigation and related technology in reducing outliers in coronal and sagittal prosthesis alignments, the error in axial rotation has not been reduced [38, 39]. Mahoney et al. revealed that the robotic-arm assisted TKA using MAKO (MAKO: robotic interactive orthopedic arm system, Stryker, Fort Lauderdale, Florida, USA) demonstrated greater accuracy for tibial component alignment, femoral component rotation and tibial slope and provided greater 3D accuracy to plan for various component positioning parameters [16].

In this study, the rotational angle for the femoral component was 2.4 ± 2.6° and 9.2 ± 2.6° for the RA group and Manual group cohorts, respectively (*p* < 0.01), and RA-TKA reduced the rotational mismatch outliers between femoral and tibial components compared to Conv-TKA (*p* < 0.01).

Kono et al. revealed that femoral component in low-PROMs group had more axial external rotation than did that in high-PROMs group after BCS-TKA [40]. We have previously reported that achieving tightness in the medial gap and looseness in the lateral gap at 90° of flexion results in improved postoperative Patient-Reported Outcome Measures (PROMs) when utilizing Journey II BCS with a balancer [21, 27]. Therefore, the RA surgical technique, which determines prosthetic alignment by considering the soft tissue envelope and incorporates an intraoperative joint-balancing procedure, has shown greater reduction in outliers in rotational alignment of the femoral component and less rotational mismatch between the femoral and tibial components compared to the Manual group.

Fujita et al. demonstrated that rotational mismatch between the femoral and tibial components and the postoperative clinical outcomes at one year postoperatively, including flexion angle, objective indicators, functional activity scores and total 2011 KSS score, were negatively correlated, and therefore when a BCS system is used for conventional TKA, surgeons should avoid excessive rotational mismatch [41].

**Fig. 4:**
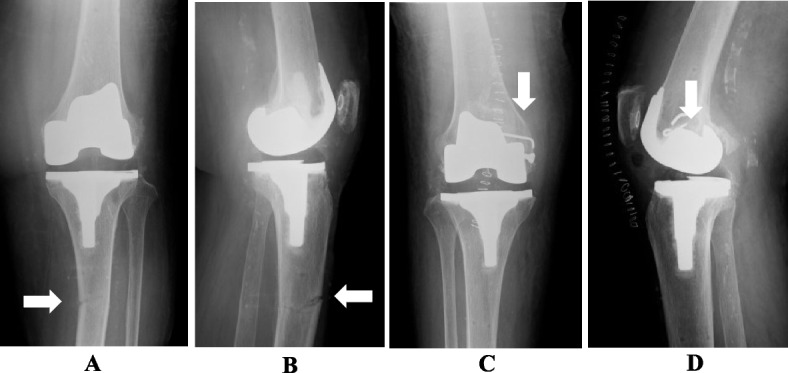
Intra and postoperative fractures during robotic assisted total knee arthroplasty using bury pin threads (4.0 mm). **A**, **B** 78-year-old woman in cast immobilization for tibial tibial diaphysis fracture (white arrow) at 1 month after surgery, **C**, **D** 89-year-old woman with intraoperative fracture of medial condyle of femur (white arrow) with solid screw and anchor fixation. **A**, **C** AP view. **B**, **D** lateral view

In this study, the RA group did not show any improvement in FJS-12 scores at 1 to 2 years postoperatively compared to the Manual group. The reason was that several patients could not forget the discomfort of the belly pin thread insertion site; Navio and CORI used a thicker 4 mm bury pin threads, while the other semi-active RA techniques used a 3.2 mm bury pin thread (Table 5). East Asia have short height and small morphology, as a result, two patients developed intraoperative and postoperative iatrogenic fractures due to bury pin threads (Fig. 4).

**Table 5:**
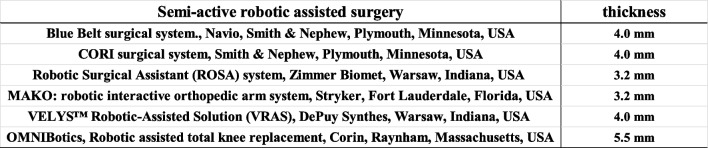
The thickness of berry pin thread thickness in semi-active robotic assisted surgery

Carlson et al. revealed that patients can expect marked improvement in the FJS-12 score during the first year after Conv-TKA, followed by slight continued improvement between 2 and 3 years, and a decline after 4 years [42]. In a multicenter study using Mako surgical system, Joo et al. reported that there were significant gradual improvements in PROMs from baseline preoperatively to 1–2 years and then to > 2 years of follow-up [43]. On the other hand, RA-TKA with Navio achieved a better FJS-12 score at 2 years postoperatively than Conv-TKA [23]. Based on these results, we expect that the results of this study will also show positive longitudinal changes in the FJS-12.

Our study has some limitations. First, the sample size was small and the follow-up period was short. In addition, the subjects enrolled only patients with primary osteoarthritis of the knee with varus deformity. Therefore, the results of the present study are not necessarily applicable to other diseases and deformities. Second, 3DCT measurements were taken in the supine position, so the lower limb alignment under weight-bearing conditions was not measured. In the pre- and postoperative supine position, a plate for dorsalis flexing of the ankle joint is placed on the sole of the foot [12, 22, 26, 27]. Fourth, when determining the soft tissue balance of individual patients in RA technique, the surgeon should be aware that the osteophytes of medial posterior femoral condyle and medial posterior tibial plateau will affect the postoperative soft tissue balance with MJG in extension and flexion. Future larger prospective studies with mid- and long-term PROMs are required to further substantiate our findings.

The clinical relevance of the present study is that validated 3DCT measurement showed that an image-free RA-TKA system reduced the outliers of rotational mismatch between femoral and tibial components, compared to Conv-TKA, but RA-TKA did not improve FJS-12 compared to Conv-TKA between 1 and 2 years postoperatively.

## Data Availability

The datasets used and analysed during the current study are available from the corresponding author.
